# Symptomatic Infection is Associated with Prolonged Duration of Viral Shedding in Mild Coronavirus Disease 2019: A Retrospective Study of 110 Children in Wuhan

**DOI:** 10.1097/INF.0000000000002729

**Published:** 2020-06-05

**Authors:** Yingying Lu, Yi Li, Wenyue Deng, Mingyang Liu, Yuanzhi He, Lingyue Huang, Mengxue Lv, Jianxin Li, Hao Du

**Affiliations:** From the * Department of Neurosurgery, Wuhan Children’s Hospital (Wuhan Maternal and Child Healthcare Hospital), Tongji Medical College, Huazhong University of Science and Technology, Wuhan, China; † Department of Hematology and Oncology, Wuhan Children’s Hospital (Wuhan Maternal and Child Healthcare Hospital), Tongji Medical College, Huazhong University of Science and Technology, Wuhan, China; ‡ Department of Nerve Electrophysiology, Wuhan Children’s Hospital (Wuhan Maternal and Child Healthcare Hospital), Tongji Medical College, Huazhong University of Science and Technology, Wuhan, China

**Keywords:** coronavirus disease 2019, children, symptomatic infection, viral shedding, pneumonia

## Abstract

Information regarding viral shedding in children with coronavirus disease 2019 (COVID-19) was limited. This study aims to investigate the clinical and laboratory characteristics associated with viral shedding in children with mild COVID-19.

**Methods:**

The clinical and laboratory information of 110 children with COVID-19 at Wuhan Children’s Hospital, Wuhan, China, from January 30 to March 10, 2020, were analyzed retrospectively.

**Results:**

The median age was 6 years old. The median period of viral shedding of COVID-19 was 15 days (interquartile range [IQR], 11–20 days) as measured from illness onset to discharge. This period was shorter in asymptomatic patients (26.4%) compared with symptomatic patients (73.6%) (11 days vs. 17 days). Multivariable regression analysis showed increased odds of symptomatic infection was associated with age <6 years (odds ratio [OR] 8.94, 95% confidence interval [CI]: 2.55–31.35; *P* = 0.001), hypersensitive C-reactive protein >3.0 mg/L (OR 4.89; 95% CI: 1.10–21.75; *P* = 0.037) and presenting pneumonia in chest radiologic findings (OR 8.45; 95% CI: 2.69–26.61; *P* < 0.001). Kaplan-Meier analysis displayed symptomatic infection (*P* < 0.001), fever (*P* = 0.006), pneumonia (*P* = 0.003) and lymphocyte counts <2.0 × 10^9^/L (*P* = 0.008) in children with COVID-19 were associated with prolonged duration of viral shedding in children with COVID-19.

**Conclusion:**

Prolonged duration of viral shedding in children with COVID-19 was associated with symptomatic infection, fever, pneumonia and lymphocyte count less than 2.0 × 10^9^/L. Monitoring of symptoms could help to know the viral shedding in children with COVID-19.

## Abstract 2

Supplemental Digital Content is available in the text.

An outbreak of coronavirus disease 2019 (COVID-19) caused by severe acute respiratory syndrome coronavirus 2 (SARS-CoV-2) has rapidly spread around the world.^1^ The clinical manifestations of SARS-CoV-2 infection in pediatric patients are classified as mild asymptomatic infection, ordinary symptomatic pneumonia, severe pneumonia, and critical cases with high mortality.^2^

Children with COVID-19 have a relatively milder clinical course than infected adults.^3,4^ Severe and critical cases of infection with SARS-CoV-2 in children have been described,^5^ but the mild and ordinary case data are lacking. Information regarding viral shedding in children with mild and ordinary forms of COVID-19 is important for the optimization of treatment and prevention of transmission of the disease.

Published studies have reported that high viral loads of SARS-CoV-2 can be detected soon after the onset of symptoms in patients with COVID-19.^6^ However, studies on the duration of negative conversion of SARS-CoV-2 in throat or nasopharyngeal swab specimens in children with COVID-19 are scarce. Therefore, this study aimed to summarize the clinical and laboratory information associated with viral shedding in children with mild and ordinary COVID-19.

## MATERIALS AND METHODS

### Patients and Laboratory Confirmation of SARS-CoV-2

This study enrolled 110 children with mild and ordinary COVID-19 who were hospitalized in the confirmed-infection isolation wards at Wuhan Children’s Hospital (Wuhan Maternal and Child Healthcare Hospital), Tongji Medical College, Huazhong University of Science and Technology, Wuhan, China from January 30, 2020, to March 10, 2020. The inclusion criteria were that patients were as follows: (1) positive for SARS-CoV-2 RNA after the analysis of throat or nasopharyngeal swab specimens using real-time reverse transcription PCR (RT-PCR) assay and (2) discharged after recovery. Exclusion criterion was patients with indefinite time of illness onset (Figure, Supplemental Digital Content 1; http://links.lww.com/INF/D945). Wuhan Children’s Hospital is assigned by the government as the only hospital responsible for the central treatment of pediatric patients with COVID-19 in Wuhan. All cases included in the study were diagnosed according to the WHO Interim Guidelines and National Recommendations for Diagnosis and Treatment of COVID-19 (7th edition).^2,7^ SARS-CoV-2 RNA was extracted from the respiratory specimens using the High Pure Viral Nucleic Acid Kit (Zhongzhi, Wuhan, China), and the diagnostic criteria of the RT-PCR assay were based on the protocol by WHO.^8^ This study was approved by the Medical Ethics Committee of Wuhan Children’s Hospital, Tongji Medical College, Huazhong University of Science and Technology, and was carried out in accordance with the Declaration of Helsinki.

### Data Collection

The demographic, clinical, laboratory, radiologic and therapeutic information of the patients were obtained from electronic medical records. Laboratory results included routine blood examination, coagulation function tests, blood biochemistry, lymphocyte subsets (T cells, B cells and natural killer cells), cytokines (interleukin [IL]-2, IL-4, IL-6, IL-10, tumor necrosis factor [TNF]-α and interferon [IFN]-γ) and inflammatory factors (hypersensitive C-reactive protein [hs-CRP], ferritin and procalcitonin) on admission. Chest radiologic findings were obtained on admission or the most obvious findings during hospitalization. The dates of illness onset, last exposure, diagnosis and discharge of patients were also recorded. Inpatients were discharge when they met the appropriate criteria,^2^ one of which was testing negative for SARS-CoV-2 RNA by a throat or nasopharyngeal swab specimens two continuous times (with an interval time of more than 24 hours). Thus, we regarded the time from illness onset to discharge as the duration of viral shedding of SARS-CoV-2 in symptomatic children. Asymptomatic children came to the hospital due to an exposure history or abnormal chest radiologic imaging. Therefore, we regarded the time from dates of last exposure or abnormal chest radiologic imaging to discharge as the duration of viral shedding in asymptomatic children.

### Statistical Analysis

Categoric variables were displayed as numbers and percentages. Continuous variables were presented as median (interquartile range [IQR]) and were compared using independent group *t*-tests when the data were distributed normally. The Mann-Whitney test was used when the data were not normally distributed. Categoric variables were compared using the *χ*^2^ test or Fisher’s exact test when the data were limited. Univariate and multivariate analyses with stepwise logistic regression were performed to identify risk factors for symptomatic infection. The cutoff point was selected from the median or quartile of each candidate risk factor. Prolonged duration of viral shedding was analyzed by the Kaplan-Meier method and the stratified log-rank statistic in different groups. Symptoms, abnormal laboratory markers, and pneumonia were used as potential factors affecting the duration of viral shedding, and the negative conversion of SARS-CoV-2 was selected as the event endpoint. Statistical analyses were performed using SPSS Software version 23.0. Statistical significance was defined as a two-sided *P*-value of less than 0.05.

## RESULTS

### Demographic and Clinical Characteristics

A total of 110 children with mild or ordinary COVID-19 were included in the current study, including 81 (73.6%) symptomatic patients and 29 (26.4%) asymptomatic patients. The median age of these patients was 6 years (range, 2 months to 15 years). An approximately 1.2:1.0 male-to-female ratio was found. In symptomatic patients, the most common symptoms were cough and dyspnea (51.8%), followed by fever (50.9%). Notably, 26 (23.6%) patients had digestive symptoms. All patients were administered antiviral therapy, of which interferon-α nebulization was the most frequently used. None of the patients required oxygen therapy. Detailed information is shown in Table 1.

**TABLE 1.**
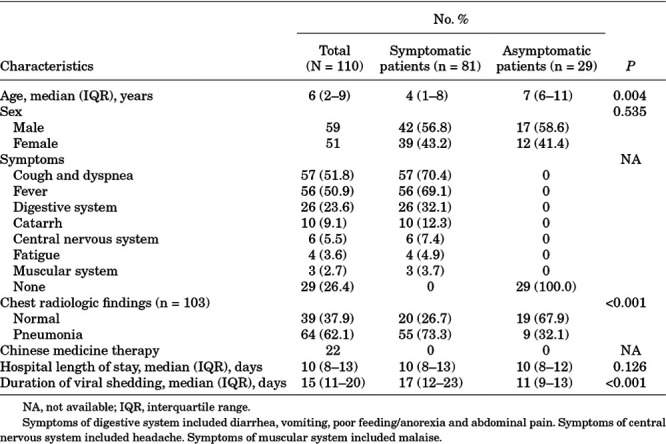
Demographic and Clinical Characteristics

### Laboratory Features

Results from all patients were recorded on the date of admission to the hospital. Dynamic changes in laboratory findings were not performed because no deterioration of manifestations or radiologic results was observed in these patients. In all patients, a slightly increased level of neutrophils (8.2%), D-dimer (12.4%), hs-CRP (19.1%), procalcitonin (47.3%), IL-6 (8.2%), IL-10 (13.7%) and slightly decreased levels of neutrophils (7.3%), fibrinogen (37.8%), globulin (27.3%), CD4+/CD8+ T cell ratio (19.6%) and natural killer cells (26.1%) were observed (Table 2). We compared laboratory results between symptomatic patients and asymptomatic patients and found no difference except for slight anemia (*P* = 0.022), and slightly increased levels of aspartate aminotransferase (*P* = 0.004), which were observed only in symptomatic patients (Table 2).

**TABLE 2.**
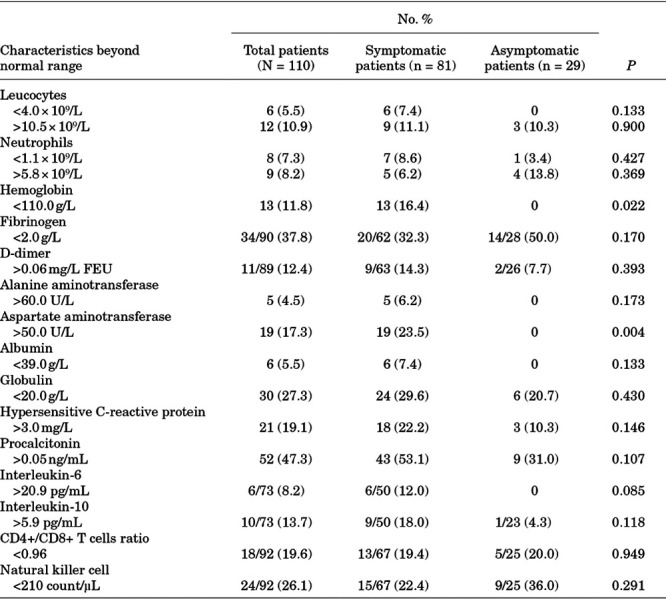
Abnormal Laboratory Results

### Risk Factors Associated with Prolonged Viral Shedding

**FIGURE 1.**
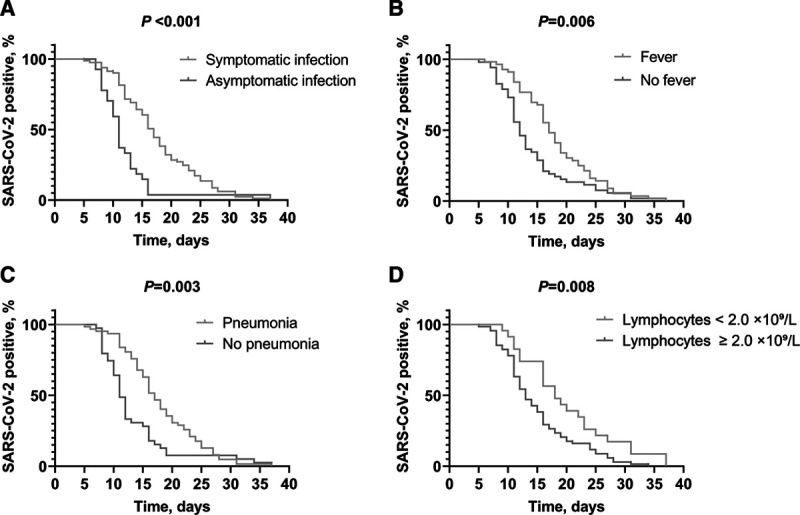
Factors associated with prolonged duration of viral shedding in children with COVID-19. Figure shows symptomatic infection (A), fever (B), pneumonia (C) and lymphocytes <2.0 × 10^9^/L (D) were associated with prolonged duration of viral shedding in Children with COVID-19. Data were analyzed by the Kaplan-Meier method and negative conversion of SARS-CoV-2 was selected as the event endpoint. COVID-19, coronavirus disease 2019; SARS-CoV-2, severe acute respiratory syndrome coronavirus 2.

The median duration of viral RNA shedding was 15 days (IQR 11–20 days), ranging from 5 to 37 days. The duration of viral shedding in symptomatic patients (17 [IQR 12–23]) was longer than that in asymptomatic patients (11 [IQR 9–13]). In Kaplan-Meier analysis, median age, symptoms, pneumonia, lymphocyte quartile and upper or lower range limits of laboratory markers were used as cutoff points for potential risk factors associated with prolonged duration of viral shedding. It was found that symptomatic infection (*P* < 0.001), fever (*P* = 0.006), pneumonia (*P* = 0.003) and lymphocyte counts <2.0 × 10^9^/L (*P* = 0.008) were associated with prolonged duration of viral shedding in children with COVID-19 (Fig. 1).

### Relationship Between Symptomatic Infection and Clinical Features

It was found that age younger than 6 years, lymphocyte counts <2.0 × 10^9^/L, globulin < 20.0 g/L, hs-CRP > 3.0 mg/L, procalcitonin > 0.05 ng/mL, IL-10 > 5.9 pg/mL, CD4+/CD8+ T cell ratio < 0.96 and pneumonia were associated with symptomatic infection in univariate analysis. In multivariate analysis, age younger than 6 years, hs-CRP beyond normal and pneumonia were independent risk factors for symptomatic infection in children with COVID-19 (Table 3). There were 64 (62.1%) patients who presented with pneumonia and these were more common in symptomatic patients (*P* < 0.001). The association between symptoms and pneumonia was evaluated using univariate analysis. It was found that symptomatic infection, fever, cough, dyspnea and digestive symptoms were correlated with pneumonia among children with COVID-19 (Table 4). Nevertheless, multivariate analysis showed no significant difference.

**TABLE 3.**
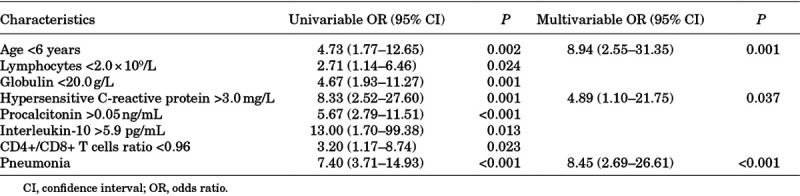
Univariate and Multivariate Analysis of Factors Associated with Symptomatic Patients

**TABLE 4.**
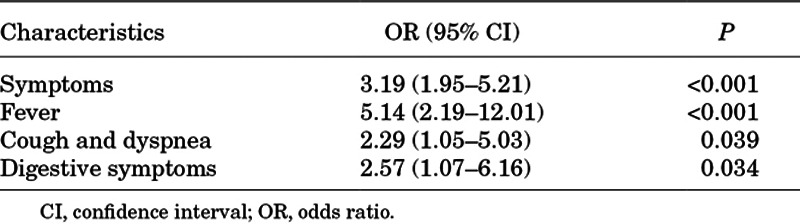
Univariate Analysis of Symptoms Associated with Pneumonia

## DISCUSSION

In the present study, the median duration of SARS-CoV-2 RNA shedding was 15 days. Prolonged viral shedding was associated with fever, pneumonia and lymphocyte counts < 2.0 × 10^9^ in children with a mild and ordinary type of COVID-19. In addition, symptomatic infection was associated with an increased risk of pneumonia. Furthermore, younger age (<6 years) was an independent risk factor for symptomatic infection in children with COVID-19.

In the current study, all the asymptomatic children at admission were mild or ordinary COVID-19, which is consistent with asymptomatic adults.^9^ Upper respiratory specimens have shown higher viral loads in symptomatic adult patients soon after symptom onset, which gradually decreased.^6^ In the present study, the duration of viral shedding in symptomatic children was significantly longer than in asymptomatic cases, which indicated that symptomatic patients had much more transmission potential and may need to be isolated.

In reports of adult inpatients with COVID-19, most of them were symptomatic and presented with radiologic evidence of pneumonia.^10,11^ Invasive mechanical ventilation was associated with prolonged viral shedding in adult patients with SARS-CoV-2 infection.^12^ In addition, higher viral RNA load and longer viral shedding of influenza A can be detected in lower respiratory tract specimens compared with upper respiratory tract specimens.^13^ Therefore, patients with pneumonia might have a high viral load in lower respiratory tract specimens, and have longer for viral clearance times.

In this study, symptomatic infection was more common in younger children. For young children who had an exposure history and presented fever, it is important to perform throat or nasopharyngeal swabs to confirm SARS-CoV-2 infection. It is worth noting that digestive symptoms such as diarrhea, vomiting, poor feeding and abdominal pain were not rare in children with COVID-19, which should not be ignored by parents and doctors. Children with COVID-19 experienced a milder clinical course than adults, and the majority of children recovered with interferon-α nebulization, similar to children with SARS.^14,15^

There are some limitations to this study. First, this was a retrospective study, and not all tests were performed and monitored during hospitalization in all patients. Second, all patients were diagnosed by throat or nasopharyngeal specimens using RT-PCR, but other specimens such as feces and urine were not monitored for SARS-CoV-2. Lastly, the ability to associate symptoms with duration of viral shedding might be limited by the sample size.

## CONCLUSIONS

We identified that prolonged duration of viral shedding was associated with fever, pneumonia and lymphocyte counts less than 2.0 × 10^9^/L in children with mild and ordinary COVID-19. Furthermore, younger age, increased hs-CRP and pneumonia were independent risk factors for symptomatic infection in children with COVID-19. Our results suggested that the clinical presentation was associated with the duration of viral shedding in children with COVID-19.

## ACKNOWLEDGEMENTS

The authors thank all the healthcare providers at the confirmed isolation wards and technical assistance of the technicians in Wuhan Children’s Hospital.

## Supplementary Material


