# Supplementary figures and images for: Symptomatic Infection is Associated with Prolonged Duration of Viral Shedding in Mild Coronavirus Disease 2019: A Retrospective Study of 110 Children in Wuhan
Source: Pediatr Infect Dis J. 2020 Jun 5;39(7):e95–9. doi: 10.1097/INF.0000000000002729 (PMC7279058; doi:10.1097/INF.0000000000002729)

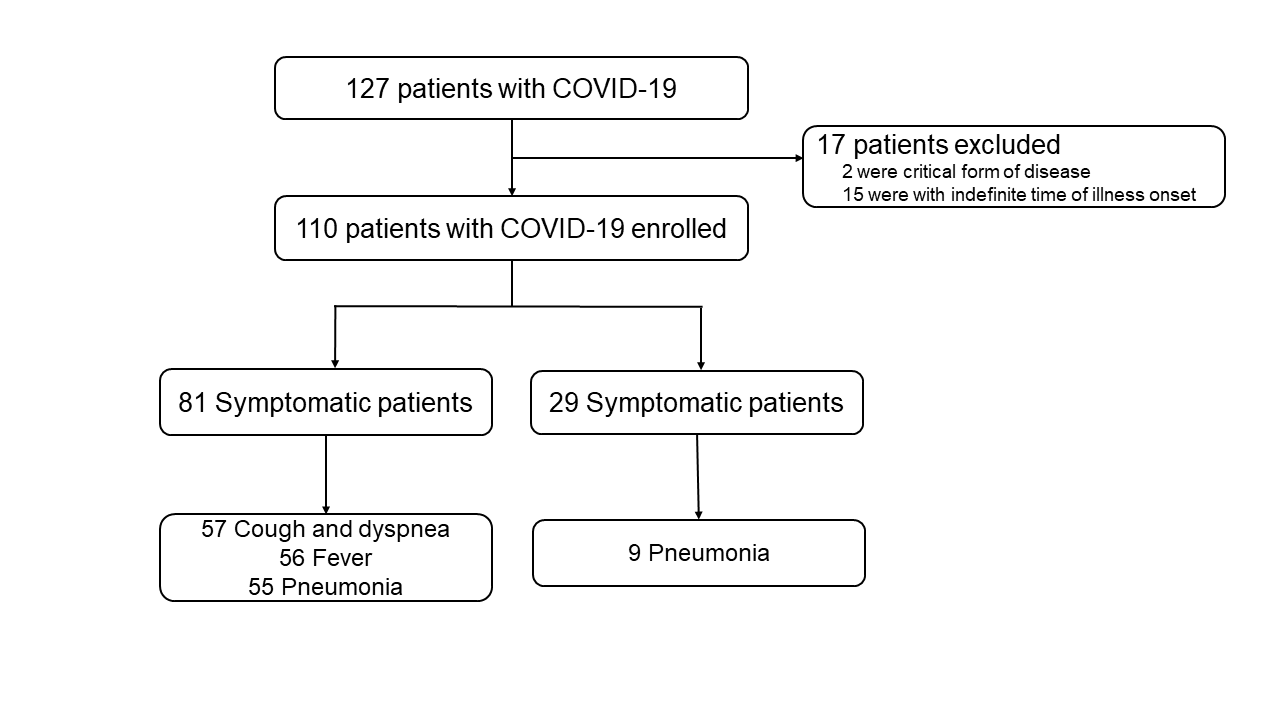

Supplement: Supplementary file 1 [file inf-39-0e95-s001.tif]
